# Gender-Dependent Phenotype in Polycystic Kidney Disease Is Determined by Differential Intracellular Ca^2+^ Signals

**DOI:** 10.3390/ijms22116019

**Published:** 2021-06-02

**Authors:** Khaoula Talbi, Inês Cabrita, Rainer Schreiber, Karl Kunzelmann

**Affiliations:** Physiological Institute, University of Regensburg, University Street 31, D-93053 Regensburg, Germany; khaoula.talbi@vkl.uni-regensburg.de (K.T.); ines.cabrita@vkl.uni-regensburg.de (I.C.); rainer.schreiber@vkl.uni-regensburg.de (R.S.)

**Keywords:** TMEM16A, ADPKD, polycystic kidneys, androgen, estrogen, CFTR

## Abstract

Autosomal dominant polycystic kidney disease (ADPKD) is caused by loss of function of PKD1 (polycystin 1) or PKD2 (polycystin 2). The Ca^2+^-activated Cl^−^ channel TMEM16A has a central role in ADPKD. Expression and function of TMEM16A is upregulated in ADPKD which causes enhanced intracellular Ca^2+^ signaling, cell proliferation, and ion secretion. We analyzed kidneys from Pkd1 knockout mice and found a more pronounced phenotype in males compared to females, despite similar levels of expression for renal tubular TMEM16A. Cell proliferation, which is known to be enhanced with loss of Pkd1^−/−^, was larger in male when compared to female Pkd1^−/−^ cells. This was paralleled by higher basal intracellular Ca^2+^ concentrations in primary renal epithelial cells isolated from Pkd1^−/−^ males. The results suggest enhanced intracellular Ca^2+^ levels contributing to augmented cell proliferation and cyst development in male kidneys. Enhanced resting Ca^2+^ also caused larger basal chloride currents in male primary cells, as detected in patch clamp recordings. Incubation of mouse primary cells, mCCDcl1 collecting duct cells or M1 collecting duct cells with dihydrotestosterone (DHT) enhanced basal Ca^2+^ levels and increased basal and ATP-stimulated TMEM16A chloride currents. Taken together, the more severe cystic phenotype in males is likely to be caused by enhanced cell proliferation, possibly due to enhanced basal and ATP-induced intracellular Ca^2+^ levels, leading to enhanced TMEM16A currents. Augmented Ca^2+^ signaling is possibly due to enhanced expression of Ca^2+^ transporting/regulating proteins.

## 1. Introduction

Male gender is a risk factor for progression of autosomal-dominant polycystic kidney disease (ADPKD) [1,2]. Affected men demonstrate faster loss of renal function and earlier onset of end stage renal disease, when compared to women [3]. Orchiectomy led to reduced renal size and cyst volume density, indicating attenuation of renal disease. In contrast, testosterone substitution was shown to antagonize the protective effect of gonadal ablation [4]. Moreover, in females, testosterone increased kidney size and cyst growth, clearly identifying androgens as a progression factor. On the other hand, estrogens have been proposed as protective hormones [5]. These results have been confirmed in additional experiments with rats [6,7]. Thus both, androgens and estrogens have an impact on cyst growth and disease progression in ADPKD.

Evidence has been provided for a crucial role of two chloride ion channels in the pathology of ADPKD, namely protein kinase A-regulated cystic fibrosis transmembrane conductance regulator (CFTR) and the Ca^2+^-activated Cl^−^ channel transmembrane 16A (TMEM16A). Support for a pro-secretory role of CFTR in ADPKD came from a number of in vitro studies [8,9,10,11], ex vivo experiments in embryonic renal cysts in metanephric organ culture [12,13], and observations in vivo in animals and humans [12,14,15]. In contrast, another in vivo study could not confirm a protective effect of missing CFTR-function in cystic fibrosis for ADPKD [16]. 

Subsequent work identified a contribution of purinergic Ca^2+^ signaling to ADPKD [17,18,19]. In a series of studies in vitro, in metanephric renal organ cultures, and in mice in vivo, we demonstrated the essential contribution of TMEM16A to renal cyst formation in ADPKD [20,21,22,23,24]. We showed that knockout of Tmem16a or inhibition of TMEM16A in vivo by the FDA-approved drugs such as niclosamide, benzbromarone, and the TMEM16A-specific inhibitor Ani9 largely reduced cyst enlargement and abnormal cyst cell proliferation. Based on these results, we proposed a novel therapeutic concept for the treatment of ADPKD, based on the inhibition of TMEM16A.

In the present study we asked whether enhanced expression or function of TMEM16A, and/or hormonal regulation may account for the more severe phenotype in male ADPKD. Typically, loss of PKD1 leads to a more severe phenotype than loss of PKD2 and accounts for about 85% of all ADPKD. We therefore examined Pkd1^−/−^ animals in the present study. While loss of PKD1 does not affect expression of PKD2, loss of either PKD1 or PKD2 leads to similar changes in intracellular Ca^2+^ signaling [23,25].

In a previous study, androgen-response elements were found in the TMEM16A promoter region, and were shown to be relevant for testosterone-dependent induction of TMEM16A [26]. Moreover, TMEM16A expression was found to be enhanced in male when compared to female sympathetic ganglia [27], but lower levels were detected in male than in female urethral smooth muscle [28]. Apart from the differences in TMEM16A-expression, CFTR might be expressed at lower levels in female ADPKD individuals, which could contribute to reduced renal cyst growth in females. In fact, a so-called cystic fibrosis (CF) gender gap describes the higher mortality in females with CF, due to lower expression of CFTR [29]. Lower CFTR-expression may be caused by estrogen-dependent regulation of CFTR [30,31].

In ADPKD, cysts occur in different renal tubular segments, yet it is assumed that most cysts are derived from the collecting duct [32]. In the present study we analyzed primary epithelial cells isolated from renal medulla and mouse mCCDcl1 collecting duct cells. We compared the properties of primary cells isolated from male and female mice, and examined whether gender differences can be reproduced in the mCCDcl1 cell line by treatment with male (dihydrotestosterone) and female (estrogen) hormones. We detected enhanced renal cyst growth and cell proliferation in male mice lacking expression of the polycystic kidney disease gene Pkd1 (polcycystin 1), when compared to female Pkd1^−/−^ mice. The data suggest enhanced proliferation, increased basal Ca^2+^ levels, and larger secretory chloride currents in cells derived from male Pkd1^−/−^ kidneys, which is likely to contribute to the enhanced progression in male ADPKD patients.

## 2. Results

### 2.1. Male Mice Lacking Expression of Pkd1 Show a Larger Number of Renal Cysts, But Similar Levels of Expression of TMEM16A

**Figure 1:**
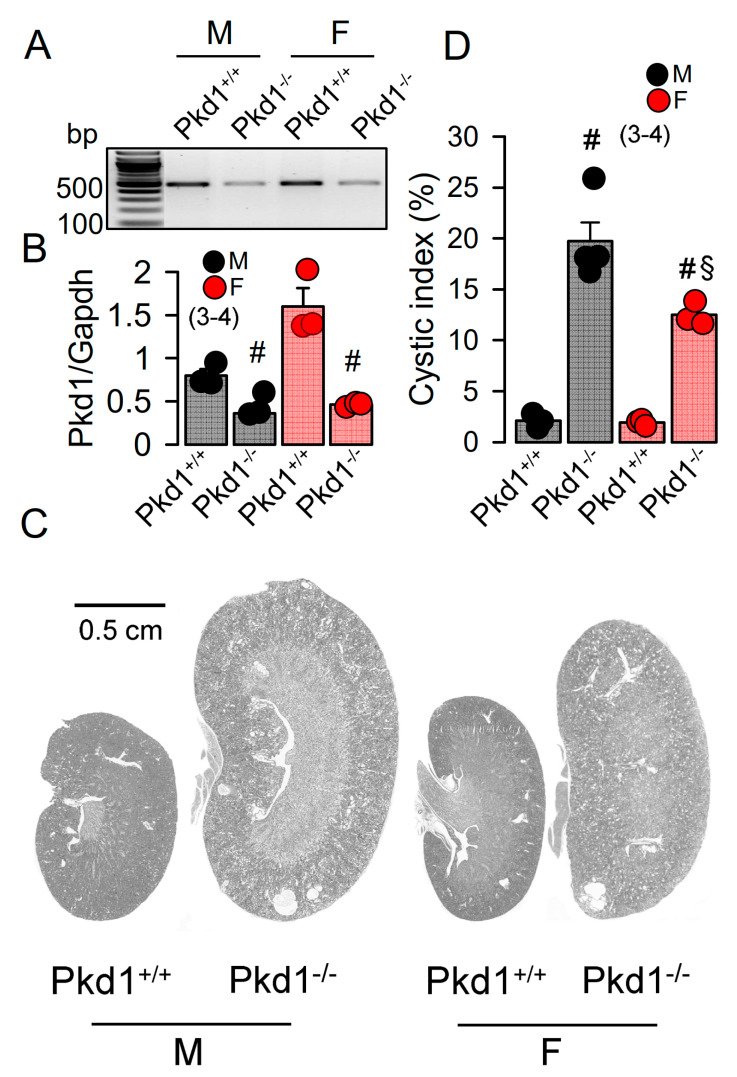
Renal cysts in male and female PKD1-knockout mice. (**A**,**B**) RT-PCR of Pkd1 in renal primary epithelial cells from male (M) and female (F) Pkd1^+/+^ and Pkd1^−/−^ mice, and semiquantitative analysis of expression. (**C**) HE staining of whole kidneys and analysis of renal cysts by stitching microscopy. Male Pkd1^−/−^ kidneys had larger sizes when compared to kidneys from female Pkd1^−/−^ mice. (**D**) Cystic index in male and female Pkd1^+/+^ and Pkd1^−/−^ kidneys. Mean ± SEM (number of animals in each series). ^#^ significant difference when compared to Pkd1^+/+^ (*p* < 0.05; unpaired t-test). ^§^ significant difference when compared to male (*p* < 0.05; ANOVA with Tukey’s post-hoc test).

The KspCreERT2;Pkd1lox;lox system was used to obtain a tamoxifen inducible tubule-specific knockout of Pkd1, as described previously [24,33]. Knockdown of Pkd1 in male and female mice was validated by RT-PCR (Figure 1A,B). Ten weeks old Pkd1^−/−^ mice demonstrated multiple renal cysts, which were more pronounced in males when compared to females (Figure 1C,D). 

**Figure 2:**
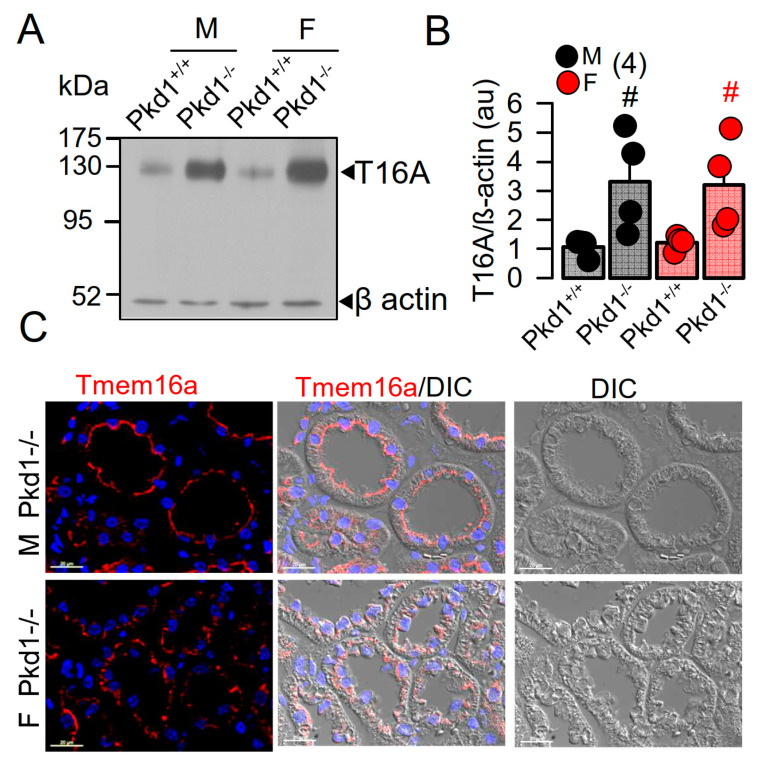
Expression of TMEM16A in male and female PKD1^−/−^ mice. (**A**,**B**) Western blotting of TMEM16A-expression in primary renal epithelial cells from kidneys of male and female Pkd1^+/+^ and Pkd1^−/−^ mice. (**C**) Immunocytochemistry of Tmem16a in kidneys from male and female Pkd1^−/−^ mice. Bar = 20 µm. Representative images from three mice each. Mean ± SEM (number of animals in each series). ^#^ significant difference when compared to Pkd1^+/+^ (*p* < 0.05; unpaired t-test).

In our previous study we reported a crucial role of the Ca^2+^-activated Cl^−^ channel TMEM16A for the development of polycystic kidneys in Pkd1^−/−^ mice and in additional in vitro models for ADPKD [20,23,24]. We therefore expected to find higher levels of TMEM16A-expression in kidneys from male Pkd1^−/−^ mice. However, both Western blotting of TMEM16A from renal lysates, as well as immunohistochemistry suggested similar levels of TMEM16A-expression in kidneys from male and female Pkd1^−/−^ mice (Figure 2). Similarly, mRNA-expression for the epithelial Cl^−^ channel cystic fibrosis transmembrane conductance regulator (CFTR) was not different between males and females (Figure S1). 

### 2.2. Cell Proliferation and Basal Ca^2+^ Levels Are More Enhanced in Renal Epithelial Cells from Pkd1^−/−^ Males Than Pkd1^−/−^ Females 

**Figure 3:**
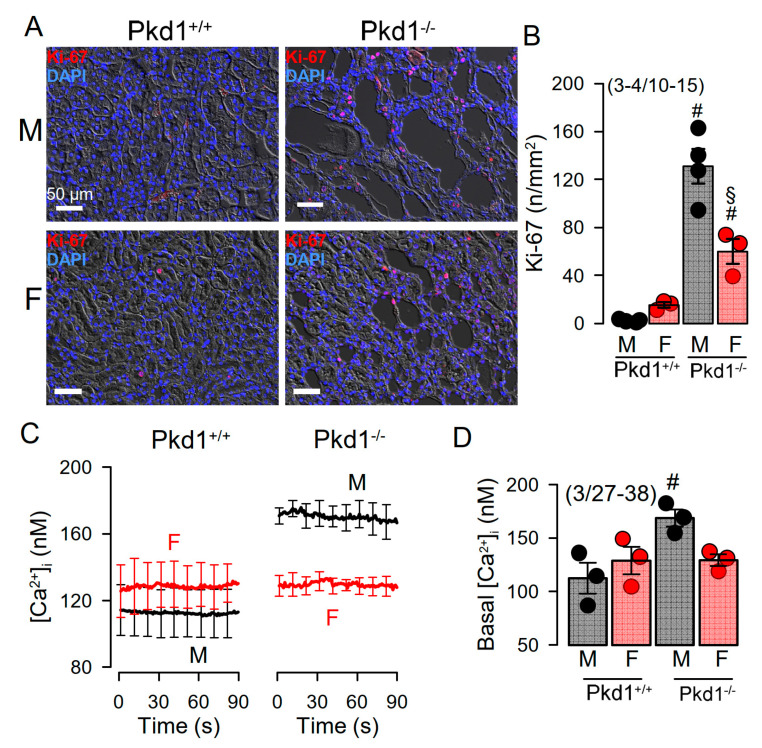
Cell proliferation and intracellular Ca^2+^ concentrations in renal epithelial cells from Pkd1^+/+^ and Pkd1^−/−^ mice. (**A**) Analysis of tubular epithelial cell proliferation in kidney sections from male and female Pkd1^+/+^ and Pkd1^−/−^ mice as indicates by Ki-67 staining. Bars = 50 µm. (**B**) Summary of Ki-67 positive cells/mm^2^ tissue area in kidneys from male and female Pkd1^+/+^ and Pkd1^−/−^ mice. Bars = 50 µm. (**C**,**D**) Intracellular basal Ca^2+^ concentrations ([Ca^2+^]_i_) as assessed by Fura2 indicates higher basal [Ca^2+^]i_I_ in male Pkd1^−/−^ mice. Mean ± SEM (number of animals/number of experiments in each series). ^#^ Significant difference when compared to Pkd1^+/+^ (*p* < 0.05; unpaired t-test). ^§^ Significant difference when compared to male (*p* < 0.05; ANOVA and Tukey’s post-hoc test).

Cell proliferation is enhanced in kidneys from Pkd1^−/−^ mice [24]. We compared the cell proliferation in males and females using Ki-67 staining. The results show that cell proliferation is enhanced in kidneys of both male and female Pkd1^−/−^ mice, however, proliferation was more enhanced in males (Figure 3A,B). Cellular Ca^2+^ levels are intimately related with cell proliferation, and have been shown to be augmented in renal epithelial cells from mice lacking expression of Pkd1, which leads to enhanced expression of TMEM16A [23,24,25]. We therefore compared the basal Ca^2+^ levels in primary renal epithelial cells from male and female mice. Remarkably, knockout of Pkd1 caused higher basal Ca^2+^ levels in renal epithelial cells from males, but not in cells from female kidneys (Figure 3C,D). These results suggest a contribution of enhanced intracellular Ca^2+^ levels to augmented cell proliferation and cyst development in male kidneys. 

**Figure 4:**
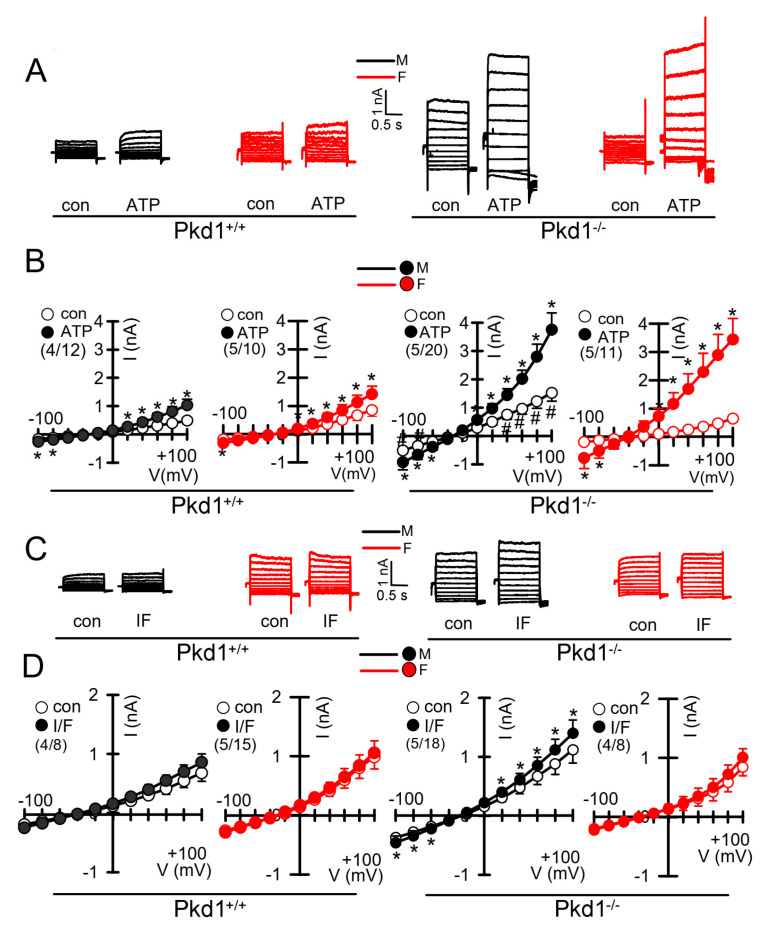
Whole cell patch clamp experiments in primary renal epithelial cells from male and female Pkd1^+/+^ and Pkd1^−/−^ mice. (**A**) Original whole cell overlay currents of basal and ATP (50 µM)—activated currents in male and female Pkd1^+/+^ and Pkd1^−/−^ mice. (**B**) Summary i/v curves of basal and ATP-activated currents indicating larger ATP-activated currents in Pkd1^−/−^ mice and larger basal currents in male Pkd1^−/−^ mice. (**C**) Original overlay currents of basal and IF (100 µM IBMX and 2 µM forskolin)—activated currents in male and female Pkd1^+/+^ and Pkd1^−/−^ mice. (**D**) Summary i/v curves of basal and IF-activated currents indicating small but significant IF-activated currents in male Pkd1^−/−^ mice. Mean ± SEM (number of animals/number of experiments in each series). * Significant activation by ATP and IF, respectively (*p* < 0.05; paired t-test). ^#^ Significant difference compared to other basal currents (*p* < 0.05; ANOVA and Tukey’s post-hoc test).

We further compared the whole cell currents measured in primary renal epithelial cells isolated from male and female kidneys. Primary cells were isolated from male and female Pkd1^+/+^ and Pkd1^−/−^ mice. As reported previously, in cells isolated from male or female Pkd1^+/+^ animals, we found little activation of TMEM16A currents by increase of intracellular Ca^2+^, using the purinergic ligand ATP (Figure 4A,B, left panels). In contrast, cells isolated from Pkd1^−/−^ animals showed large ATP-activated whole cell currents. We noticed that the basal current in male Pkd1^−/−^ cells was significantly larger than that measured in female Pkd1^−/−^ cells (Figure 4A,B, right panels). This may be explained by the fact that male Pkd1^−/−^ cells showed a higher basal intracellular Ca^2+^ concentration (Figure 3C,D). We also examined the whole cell currents activated by IBMX and forskolin (IF), which both increase intracellular cAMP. No activation of whole cell currents was observed in Pkd1^+/+^ cells, while a small but significant current was activated in male Pkd1^−/−^ cells (Figure 4C,D). Patch clamp ion current data were supported by additional iodide quenching data, which also indicated a larger halide permeability in male when compared to female Pkd1^−/−^ cells (Figure S2). Taken together, higher basal Ca^2+^ concentrations, enhanced proliferation and larger basal Cl^−^ currents may explain the more pronounced cystic phenotype in male Pkd1^−/−^ kidneys.

### 2.3. Testosterone Augments ATP-Induced Whole Cell Currents in Female Pkd1^+/+^ Cells 

**Figure 5:**
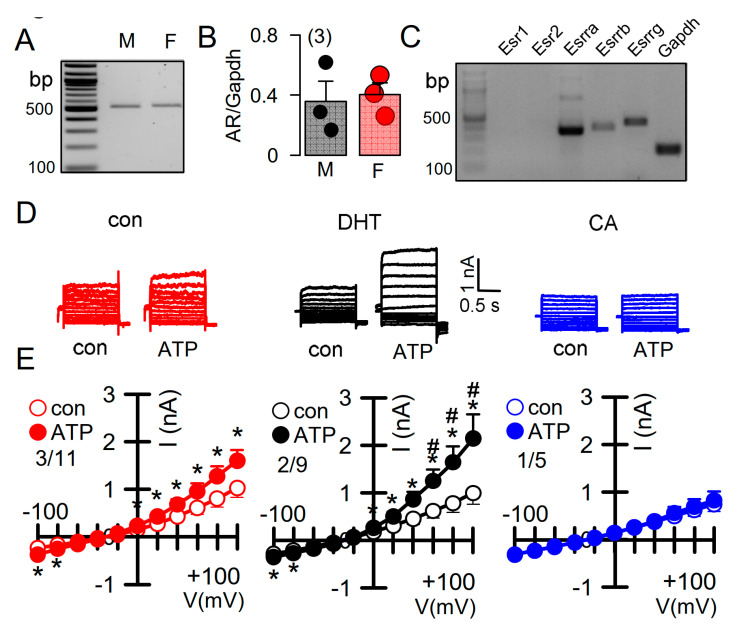
Effect of dihydrotestosterone on whole cell currents in primary renal epithelial cells from female Pkd1^+/+^ mice. (**A**,**B**) RT-PCR analysis of expression of the androgen receptor (AR) in primary renal epithelial cells from male and female kidneys. (**C**) Expression of estrogen receptors in primary renal epithelial cells from female Pkd1^+/+^ mice. (**D**,**E**) Whole cell current overlays and corresponding I/V-curves from primary renal epithelial cells of female Pkd1^+/+^ mice. ATP (50 µM) -activated currents were augmented in cells-incubated with dihydrotestosterone (DHT; 10 µM, 24 h), but were absent in cyproterone acetate (CA, 10 µM, 24 h) incubated cells. Mean ± SEM (number of animals/number of experiments in each series). * Significant activation by ATP (*p* < 0.05; paired t-test). ^#^ Significant difference when compared to con (no DHT) (*p* < 0.05; ANOVA and Tukey’s post-hoc test).

Androgens have been implicated in the severity of the disease phenotype in ADPKD [34]. We therefore examined the impact of dihydrotestosterone (DHT) on the ion currents in primary renal epithelial cells, isolated from female Pkd^+/+^ mice. Expression of androgen receptors in primary renal epithelial cells was determined by RT-PCR (Figure 5A,B). Moreover, different types of estrogen receptors were found to be expressed in these cell (Figure 5C). As expected, ATP-activated whole cell currents were moderate in control cells, but were significantly enhanced in cells treated with DHT. In contrast, in cells treated with the inhibitor of androgen receptors, cyproterone acetate (CA), no whole cell currents could be activated by ATP (Figure 5D,E). 

**Figure 6:**
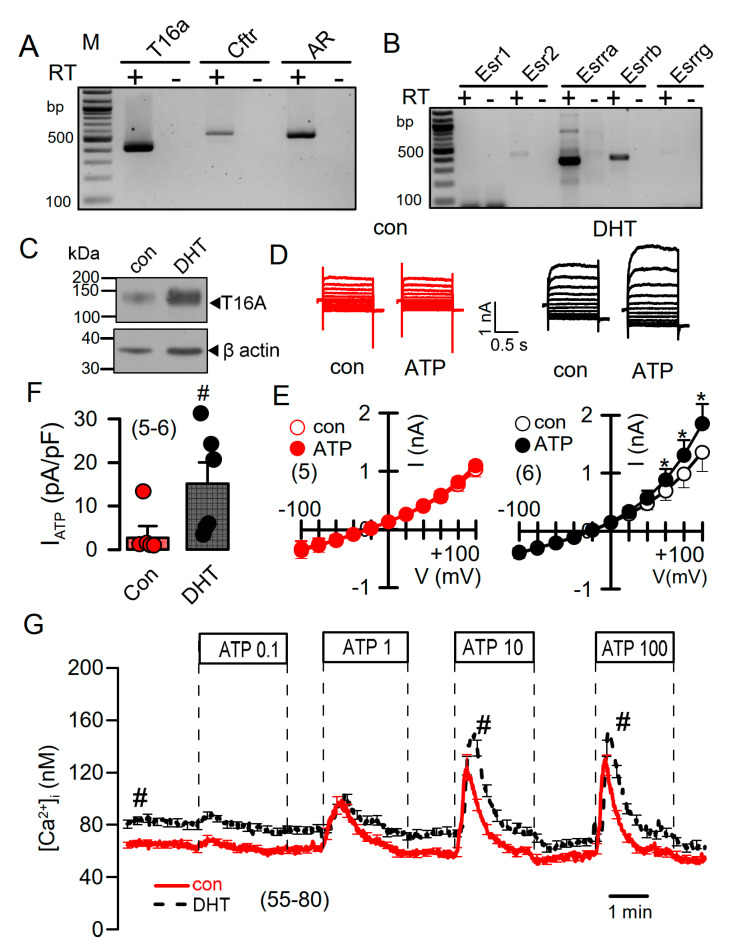
Effect of dihydrotestosterone on whole cell currents and intracellular Ca^2+^ concentrations in mouse mCCDcl1 cortical collecting duct cells. (**A**,**B**) RT-PCR analysis of expression of Tmem16a, Cftr, androgen receptors (**A**), and estrogen receptors (**B**) in Pkd1^+/+^ mCCDcl1 mouse collecting duct cells. (**C**) Western blot from mCCDcl1 cells, indicating upregulation of Tmem16a-expression by DHT. (**D**,**E**) Whole cell current overlays (**D**), corresponding I/V-curves (**E**), and summary of ATP (0.1 µM) -activated whole cell currents (**F**) from mCCDcl1 cells, indicating larger basal and ATP-activated currents in DHT (10 µM; 24 h) treated cells. Current densities (pA/pF) were assessed at the clamp voltage of +100 mV. (**G**) Basal and ATP (0.1–100 µM)–induced increase in intracellular Ca^2+^ concentration in control (con) and DHT-incubated mCCDcl1 cells. Mean ± SEM (number of experiments). * Significant activation by ATP (*p* < 0.05; paired t-test). ^#^ Significant difference compared to con (*p* < 0.05; unpaired t-test).

DHT-dependent regulation of Ca^2+^-activated Cl^−^ currents was further examined in mCCDcl1 mouse renal cortical collecting duct cells [35]. These cells express TMEM16A, CFTR, and receptors for testosterone and estrogen (Figure 6A,B). However, the main estrogene receptors Esr1 and Esr2 are not expressed in renal epithelial cells, suggesting that estrogen-dependent regulation of protein expression is not dominant in kidney. 

Treatment with DHT-enhanced expression of TMEM16A in mCCDcl1 cells (Figure 6C). In these cells, ATP concentrations as low as 0.1 µM stimulated a whole cell current that was not observed in the absence of DHT (Figure 6D–F). Upregulation of ATP-activated whole cell currents by DHT was also observed in mouse M1 collecting duct cells, which also showed a slight but detectable increase in cAMP-activated currents upon DHT treatment (Figure S3). No whole Cl^−^ currents could be activated in cells incubated with estrogen. 

### 2.4. Testosterone Enhances Intracellular Ca^2+^ Signals 

DHT increased the expression of TMEM16A and enhanced ATP-activated whole cell currents, which may be due to enhanced intracellular Ca^2+^ signaling. We measured the intracellular Ca^2+^ concentrations in control mCCDcl1 cells, and in cells treated with DHT. Cells were stimulated by different concentrations of ATP, which caused a concentration-dependent increase in intracellular Ca^2+^ concentration (Figure 6G). Notably, DHT did not further enhance transient Ca^2+^ increase induced by ATP, but shifted the basal Ca^2+^ concentration to higher values. This suggests a Ca^2+^ leakage out of the endoplasmic reticulum (ER) Ca^2+^ store, or enhanced Ca^2+^ influx through plasma membrane Ca^2+^ channels (Figure 6G). 

**Figure 7:**
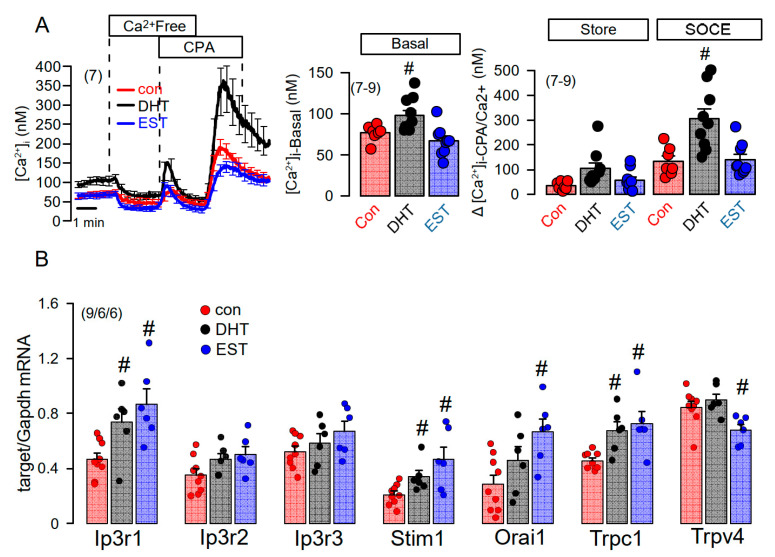
Effect of dihydrotestosterone and estrogen on intracellular Ca^2+^ concentrations and expression of Ca^2+^-regulating proteins in mouse mCCDcl1 cortical collecting duct cells. (**A**) Summary time course of the effects of extracellular Ca^2+^-free buffer and cyclopiazonic acid (10 µM) on intracellular Ca^2+^ concentrations in control mCCDcl1 cells and mCCDcl1 cells treated with dihydrotestosterone (DHT; 10 µM, 24 h) and estrogen (EST, 10 µM; 24 h). Summary of basal intracellular Ca^2+^ concentration and changes in intracellular Ca^2+^ induced by CPA (Ca^2+^ store release through leakage channels; Store) and re-addition of Ca^2+^ to the extracellular buffer (store operated Ca^2+^ entry; SOCE). (**B**) RT-PCR analysis of the expression of Ca^2+^-regulating proteins and effects of DHT and EST. Mean ± SEM (number of experiments in each series). ^#^ Significant increase when compared with con (*p* < 0.05; ANOVA).

We applied a Ca^2+^ store release protocol to empty the ER Ca^2+^ store by inhibiting the sarcoplasmic endoplasmic reticulum ATPase (SERCA), using cyclopiazonic acid (CPA). Remarkably, removal of extracellular Ca^2+^ lead to a more pronounced decrease in basal cytosolic Ca^2+^ in DHT, but not in EST-treated cells (Figure 7A, left and middle panel). Subsequent addition of CPA induced a more pronounced Ca^2+^ store release in DHT-treated cells, and a largely augmented Ca^2+^ influx upon re-addition of extracellular Ca^2+^ (Figure 7A, left and right panel). Thus, Ca^2+^ store release and store operated Ca^2+^ influx were augmented in DHT-treated cells. We analyzed the expression of a number of Ca^2+^ transporting proteins such as inositol trisphosphate receptors (IP3R1-3), the Ca^2+^ influx channels Orai1, TRPC1, TRPV4, and the ER Ca^2+^ sensor stromal interaction molecule 1 (Stim1). Notably, expression of a number of Ca^2+^ channels and Stim1 were augmented by both DHT and EST incubated cells. As shown in Figure 6C, DHT enhanced the expression of TMEM16A, while EST had no significant effect on TMEM16A-expression (data not shown). By comparing Ca^2+^ signals obtained from male and female primary renal epithelial cells, we also found higher basal Ca^2+^ levels in male cells, along with larger ATP-induced Ca^2+^ transients (Figure S4). Taken together, the more severe cystic phenotype found in males is likely to be caused by enhanced cell proliferation possibly due to enhanced basal intracellular Ca^2+^ levels, which is probably due to enhanced expression of Ca^2+^ transporting/regulating proteins.

## 3. Discussion

In the present study we analyzed the primary epithelial cells isolated from renal medulla and mouse mCCDcl1 collecting duct cells. We compared the properties of primary cells isolated from male and female mice, and examined whether gender differences can be reproduced in the mCCDcl1 cell line by treatment with DHT and EST. We investigated whether enhanced expression or function of TMEM16A, and/or hormonal regulation may account for the more severe ADPKD phenotype caused by knockout of Pkd1. As shown previously, loss of PKD1 or PKD2 led to similar changes in intracellular Ca^2+^ signaling which could be reproduced in the present study [23,25]. The present data show a higher cystic index in kidneys from male Pkd1^−/−^ mice. Although expression of the disease-associated Ca^2+^-activated Cl^−^ channel TMEM16A was not different between male and female Pkd1^−/−^ kidneys, cell proliferation, basal cytosolic Ca^2+^ levels, and basal Cl^−^ currents were larger in renal epithelial cells derived from males (Figure 1, Figure 2, Figure 3 and Figure 4). 

Proliferation of renal tubular epithelial cells is enhanced in both male and female kidneys from Pkd1^−/−^ knockout mice, however, the effect is more pronounced in male kidneys. Proliferation is predominantly due to the upregulation of TMEM16A-expression as demonstrated in our recent study [24]. TMEM16A is well-known to enhance proliferation in different types of cells including cancer cells. This is due to the upregulation of Ca^2+^ (ATP^−^) activated chloride currents and upregulated ATP-induced Ca^2+^ store release [24]. Enhanced Ca^2+^ signaling is also demonstrated by augmented store release triggered by cyclopiazonic acid with consecutive increase in store-operated Ca^2+^ entry. These changes are observed in both primary renal epithelial cells from male and female Pkd1^−/−^ mice, however, upregulation of these Ca^2+^ signals is more pronounced in epithelial cells from males (Figure S5). Furthermore, a small cAMP-regulated CFTR current was detected in the cells from male Pkd1^−/−^ kidneys, but was not found in cells from female kidneys. Expression levels for CFTR were not different between male and female kidneys as detected by semiquantitative RT-PCR or Western blotting. 

Higher TMEM16A currents and small but detectable CFTR currents were also found in renal epithelial cells upon exposure to dihydrotestosterone, which also enhanced expression of TMEM16A. Importantly, Cha et al. reported three androgen-response elements in the TMEM16A promoter region, which are relevant for the DHT-dependent induction of TMEM16A [26]. Comparable to our previous report showing inhibition of cell proliferation and renal cyst growth [24], Cha et al. reported inhibition of prostate hyperplasia by siRNA-knockdown of TMEM16A [26]. In contrast, androgens potentiated renal cell proliferation and cyst enlargement through ERK1/2-dependent and ERK1/2-independent signaling in another study [34]. However, in our study we were unable to detect the different levels for TMEM16A expression in male and female kidneys (Figure 2). A likely explanation might be that knockout of Pkd1 already induced a strong upregulation of TMEM16A-expression [24], thereby overrunning androgen-dependent regulation of expression. Notably, in cultured mCCDcl1 collecting duct cells, where Pkd1 is not knocked out, androgen-dependent upregulation of TMEM16A is detected (Figure 6).

Enhanced basal (Figure 3, Figure 6 and Figure 7) and ATP-activated (Figure 6 and Figure S4) Ca^2+^ levels were clearly detectable in male primary renal epithelial cells and androgen-treated mCCDcl1 cells. Androgens also caused increased intracellular Ca^2+^ levels in prostate and skeletal muscle cells [36,37]. A number of previous studies examined the effects of androgens on cytosolic Ca^2+^ signaling, and expression of proteins that regulate intracellular Ca^2+^ levels. Thus androgens upregulated the Ca^2+^ influx channel Orai1 in MCF-7 breast tumor cells [38], while in LNCaP cells, androgens were shown to increase cytosolic Ca^2+^ by enhancing Ca^2+^ influx through L-type channels [39], and via G-protein-coupled receptors [40,41]. Moreover, expression of the Ca^2+^ sensor in the endoplasmic reticulum, stromal interaction molecule 1 (STIM1), was shown to be regulated by androgens [42]. Conversely, estradiol was shown to inhibit the phosphorylation of STIM1 which attenuated SOCE [43]. 

We found lower expression of TRPV4 upon treatment with estradiol, which may suggest a role of TRPV4 in differential Ca^2+^ signaling in male vs. female renal epithelial cells. Notably, using RT-PCR we also found a reduced expression of TRPV4 in female Pkd1^−/−^ mice, when compared to male Pkd1^−/−^ mice (data not shown). Interestingly, enhanced expression of TRPV4 was also found in male hypertensive rats when compared to female animals [44]. However, given the number of Ca^2+^ transporting proteins that differ between cells derived from males and females, enhanced basal Ca^2+^ levels in males are likely due to a combination of differentially expressed Ca^2+^-transporting proteins. We believe that the differences in basal Ca^2+^ concentrations could be sufficient to explain the different basal activity of TMEM16A in male and female cells. Activation of TMEM16A depends largely on the membrane voltage [45]. Activation of inward currents at hyperpolarized (physiological) voltages require higher [Ca^2+^]_i_ than outward currents and are in the range of 1 µM and higher. However, Ca^2+^ concentrations related to channel activity are typically obtained by Ca^2+^-sensing dyes, which measure global cytosolic Ca^2+^ concentrations, such as Fura-2. These global [Ca^2+^]_i_ do not reflect the true Ca^2+^ levels in the TMEM16A-containing subapical compartment. We now know that TMEM16A is a membrane tether that binds the receptor for inositol trisphosphate (IP3) and thereby enhances local Ca^2+^ levels in close proximity to TMEM16A [46]. Using membrane-bound Ca^2+^ sensors, remarkably higher Ca^2+^ concentrations were found in TMEM16A-containing compartments underneath the cell membrane, causing more pronounced activation of TMEM16A [47]. Finally, differential expression of splice variants for TMEM16A could be an additional reason for the larger basal Cl^−^ currents detected in male renal epithelial cells. For example, Ferrera and collaborators reported differential Ca^2+^ sensitivity for the isoforms TMEM16A(a,b,c), and TMEM16A(a,c) [48]. Differential expression of TMEM16A isoforms should be therefore examined in subsequent studies.

The results of the present study may be summarized as follows: (i) Renal cysts found in ADPKD caused by knockout of Pkd1, are associated with enhanced basal intracellular Ca^2+^ levels and enhanced agonist (ATP)^−^ and CPA-induced Ca^2+^ store release and SOCE. (ii) The more severe phenotype in males is related to larger store release and SOCE detected in cells derived from males when compared to females (Figure S5). (iii) Stimulation of the androgen receptor by DHT in cells derived from primary female cells and in the mCCDcl1 cell line reproduces the findings obtained in primary male tissues. (iv) Gender-dependent phenotype differences are not explained by different levels of expression of the ADPKD-relevant ion channel TMEM16A. (v) Differential Ca^2+^ homeostasis in kidneys of male and female ADPKD patients is suggested. (vi) Higher Ca^2+^ levels not only enhance TMEM16A- activity, but may also contribute to higher activity of CFTR [49]. (vii) Additional work will be required to identify the molecular mechanisms underlying the gender-dependent differences of Ca^2+^ signaling in ADPKD.

## 4. Materials and Methods

### 4.1. RT-PCR

For semi-quantitative RT-PCR total RNA from primary medullary kidney epithelial cells were isolated using NucleoSpin RNA II columns (Macherey-Nagel, Düren, Germany). Total RNA (0.5 µg/25 µL reaction) was reverse-transcribed using random primer (Promega, Mannheim, Germany) and M-MLV Reverse Transcriptase RNase H Minus (Promega, Mannheim, Germany). Each RT-PCR reaction contained sense (0.5 µM) and antisense primer (0.5 µM) (Table 1), 0.5 µL cDNA and GoTaq Polymerase (Promega, Mannheim, Germany). After 2 min at 95 °C cDNA was amplified (targets 30 cycles, reference Gapdh 25 cycles) for 30 s at 95 °C, 30 s at 56 °C, and 1 min at 72 °C. PCR products were visualized by loading on Midori Green Xtra (Nippon Genetics Europe) containing agarose gels and analyzed using Image J 1.52r (NIH, Bethesda, MD, USA).

### 4.2. Cell Culture

The mouse collecting duct cell line mCCDcl1 was kindly provided by Prof. Dr. Johannes Loffing (Institute of Anatomy, University of Zurich, Zurich, Switzerland) and were grown as described previously [35]. M1 mouse collecting duct cells were grown as outlined in an earlier publication [50].

### 4.3. Renal Medullary Primary Cells Isolation

Mice were sacrificed through CO_2_ inhalation and cervical dislocation and kidneys were kept in ice-cold DMEM/F12 medium (Thermo Fisher Scientific, Darmstadt, Germany). The renal capsule was removed under sterile conditions. The medulla was separated from the cortex and chopped into smaller pieces of tissue using a sharp razor blade (Heinz Herenz, Hamburg, Germany). Tissues were incubated in Hanks balanced salt solution/DMEM/F12 (Life Technologies/Gibco^®^, Karlsruhe, Germany) containing 1 mg/ml collagenase type 2 (Worthington, Lakewood, NJ, USA) for 20 min at 37 °C. The digested tissue was passed through a 100 µm cell strainer (Merck KGaA, Darmstadt, Germany), transferred to a 50 mL falcon tube and washed with ice-cold PBS. After centrifugation at 1880 rpm for 4 min/4 °C, cells were resuspended and centrifuged 3X at 2260 rpm for 4 min at 4 °C. After washing with ice-cold PBS, tubular preparations were cultured at 37 °C/5% CO_2_ in DMEM/F12 supplemented with 1% FBS, 1% Pen/Strep, 1% L-glutamine (200 mM), 1% ITS (100×), 50 nM hydrocortisone, 5 nM triiodothyronine, and 5 nM epidermal growth factor (Sigma Aldrich, Taufkirchen, Germany). After washing with ice-cold PBS, tubular preparations were maintained at 37 °C/5% CO_2_ in DMEM/F12 supplemented with 1% FBS, 1% Pen/Strep, 1% L-glutamine (200 mM), 1% ITS (100×), 50 nM hydrocortisone, 5 nM triiodothyronine, and 5 nM epidermal growth factor (Sigma Taufkirchen, Germany). After 24 h, primary cells grew out from isolated tubules.

### 4.4. Animals and Treatments

Animal experiments were approved by the local institutional review board and all animal experiments complied with the with the United Kingdom Animals Act, 1986, and associated guidelines, EU Directive 2010/63/EU for animal experiments. Experiments were approved by the local Ethics Committee of the Government of Unterfranken/Wuerzburg (AZ: 55.2-2532-2-823). Mice with a floxed *PKD1* allele were generously provided by Prof. Dr. Dorien J.M. Peters (Department of Human Genetics, Leiden University Medical Center, Leiden, The Netherlands) [51]. Animals were hosted on a 12:12 h light:dark cycle under constant temperature (24 ± 1 °C) in standard cages. They were fed a standard diet with free access to tap water. Generation of mice with a tamoxifen inducible, kidney epithelium-specific *Pkd1*-deletion was done as previously described [24]. Mice carrying loxP-flanked conditional alleles of *Pkd1* were crossed with KSP-Cre mice in a C57BL/6 background (KspCreER^T2^; *Pkd1*^lox;lox^; abbreviated as *Pkd1*^−/−^). Mice of age 8–10 weeks were used in the experiments.

### 4.5. Histologic Analysis, Cystic Index 

Photographs from hematoxylin and eosin-stained kidney sections were taken at a magnification of ×23 and stitched to obtain a single photograph of the whole transverse kidney sections using a Axiovert 200 microscope (Zeiss, München, Germany). The whole kidney cortex was defined as the region of interest using ImageJ (version 1.48). Next, we used an algorithm (ImageJ software version 1.48, NIH, Bethesda, MD, USA) that separates normal tubule space from cystic area by defining diameters of noncystic tubules < 50 mm [32]. The whole cortex cyst area was divided by the whole cortex area and defined as the cystic index.

### 4.6. Immunohistochemistry

Five-micron thick transverse kidney sections were stained. Anti-TMEM16A (rabbit; 1:100; Abcam, Berlin, Germany) antibody was used as described previously [20]. As secondary antibodies, anti-rabbit IgG Alexa Fluor 546 (1:300; Thermo Fisher Scientific, Inc., Erlangen, Germany) was used. Immunofluorescence was detected using an Axiovert 200 microscope equipped with ApoTome and AxioVision (Zeiss, Germany).

### 4.7. Western Blotting

Proteins were isolated from perfused whole kidneys and from mCCDcl1 cells using a sample buffer containing 25 mM Tris–HCl, 150 mM NaCl, 1% Nonidet P-40, 5% glycerol, 1 mM EDTA, and 1% protease inhibitor mixture (Roche, Mannheim, Germany). Equal amounts of protein were separated using 8.5% sodium dodecyl sulfate (SDS) polyacrylamide gel. Proteins were transferred to a polyvinylidene difluoride membrane (GE Healthcare Europe GmbH, Munich, Germany) using a semi-dry transfer unit (Bio-Rad, Feldkirchen, Germany). Membranes were incubated with primary anti-TMEM16A (rabbit 1:500; Alomone, Jerusalem, Israel) mouse antibody overnight at 4 °C. Proteins were visualized using horseradish peroxidase-conjugated secondary antibody and ECL detection. Beta-Actin was used as a loading control.

### 4.8. Ki-67 Assay

Ki-67 staining was performed using a monoclonal anti-ki-67 antibody (rabbit; 1:100, Linaris, Dossenheim, Germany). Signals were amplified by the use of the Vectastain Elite ABC Kit (Vector Laboratories, Burlingame, CA, USA) according to the manufacturer’s instructions. Signals were analyzed with a Axiovert 200 microscope (Zeiss, Germany).

### 4.9. Patch Clamp

Patch-clamp experiments were performed in the fast whole-cell configuration. Patch pipettes had an input resistance of 4–6 MΩ, when filled with a cytosolic-like pipette filling solution [52] containing (mM) KCl 30, K-gluconate 95, NaH_2_PO_4_ 1.2, Na_2_HPO_4_ 4.8, EGTA 1, Ca-gluconate 0.758, MgCl_2_ 1.034, D-glucose 5, ATP 3. The pH was 7.2, and the Ca^2+^ activity was 0.1 µM. The extracellular bath perfusion was a Ringer solution containing (mmol/L) NaCl 145; KH_2_PO_4_ 0.4; K_2_HPO_4_ 1.6; glucose 5; MgCl_2_ 1; Ca^2+^-Gluconat 1.3. The access conductance was measured continuously and was 30–140 nS. Currents (voltage clamp) and voltages (current clamp) were recorded using a patch-clamp amplifier (EPC 9, List Medical Electronics, Darmstadt, Germany) and PULSE software (HEKA, Lambrecht, Germany) as well as Chart software (AD-Instruments, Spechbach, Germany). In intervals, membrane capacitance was measured using the EPC9 device. Data were stored continuously on a computer hard disc and were analyzed using PULSE software. In regular intervals, membrane voltages (*V*_c_) were clamped in steps of 20 mV from −100 to +100 mV relative to resting potential. Current densities (pA/pF) were assessed at the clamp voltage of +100 mV.

### 4.10. Iodide Quenching Experiments

For YFP-quenching assays, primary renal cells were infected with lentiviral vectors to express halide-sensitive YFP_I152L_, as previously described [53]. Primary renal cells were isolated and cultured and for each mouse 40 cells were measured. Quenching of the intracellular fluorescence generated by the iodide sensitive enhanced yellow fluorescent protein (EYFP-I152L) was used to measure the anion conductance. YFP-I152L fluorescence was excited at 500 nm using a polychromatic illumination system for microscopic fluorescence measurement (Visitron Systems, Puchheim, Germany) and the emitted light was measured at 535 ± 15 nm with a Coolsnap HQ CCD camera (Roper Scientific, Tucson, AZ, USA). Quenching of YFP-I152L fluorescence by I^−^ influx was induced by replacing 5 mM extracellular Cl^−^ with I^−^. Cells were grown on coverslips and mounted in a thermostatically controlled imaging chamber maintained at 37 °C. Cells were continuously perfused at 8 mL/min with Ringer solution and exposed to I^−^ concentration of 5 mM by replacing same amount of NaCl with equimolar NaI. Background fluorescence was subtracted, while auto-fluorescence was negligible. Changes in fluorescence induced by I^−^ are expressed as initial rates of maximal fluorescence decrease (Δ*F*/Δ*t*). For quantitative analysis, cells with low or excessively high fluorescence were discarded.

### 4.11. Measurement of [Ca^2+^]_i_

Cells were loaded with 2 µM Fura-2/AM and 0.02% Pluronic F-127 (Invitrogen, Darmstadt, Germany) in Ringer solution (mmol/L: NaCl 145; KH_2_PO_4_ 0.4; K_2_HPO_4_ 1.6; Glucose 5; MgCl_2_ 1; Ca^2+^-Gluconat 1.3) for 1 h in the dark at room temperature. Cells were perfused with Ringer solution at 37 °C and fluorescence was detected using an inverted microscope (Axiovert S100, Zeiss, Germany) and a high-speed polychromator system (VisiChrome, Puchheim, Germany). Fura-2 was excited at 340/380 nm, and emission was recorded between 470 and 550 nm using a CoolSnap camera (CoolSnap HQ, Roper Scientific, Tucson, AZ, USA). [Ca^2+^]*_i_* was calculated from the 340/380 nm fluorescence ratio after background subtraction. The formula used to calculate [Ca^2+^]*_i_* was [Ca^2+^]*_i_* = *Kd* × (*R* − *R*_min_)/(*R*_max_ − *R*) × (*S*_f2_/*S*_b2_), where *R* is the observed fluorescence ratio. The values *R*_max_ and *R*_min_ (maximum and minimum ratios) and the constant *S*_f2_/*S*_b2_ (fluorescence of free and Ca^2+^-bound Fura-2 at 380 nm) were calculated using 1 µmol/L ionomycin (Calbiochem, Merck, Darmstadt, Germany), 5 µmol/L nigericin, 10 µmol/L monensin (Sigma Aldrich, Taufkirchen, German), and 5 mmol/L EGTA to equilibrate intracellular and extracellular Ca^2+^ in intact Fura-2-loaded cells. The dissociation constant for the Fura-2•Ca^2+^ complex was taken as 224 nmol/L.

### 4.12. Materials and Statistical Analysis

All compounds used were of highest available grade of purity. Data are reported as mean ± SEM. Student’s *t* test for unpaired samples and ANOVA were used for statistical analysis. *p* < 0.05 was accepted as the significant difference. Data are expressed as mean ± SEM. Differences among groups were analyzed using one-way ANOVA, followed by a Bonferroni test for multiple comparisons. An unpaired *t* test was applied to compare the differences between the two groups. 

## Figures and Tables

**Table 1:** Primers used for PCR-analysis.

Gene Accession Number	Primer	Size (bp)
Tmem16a NM_001242349.2	s: 5′-GTGACAAGACCTGCAGCTAC as: 5′-GCTGCAGCTGTGGAGATTC	406
Cftr NM_021050.2	s: 5′-GAATCCCCAGCTTATCCACG as: 5′-CTTCACCATCATCTTCCCTAG	544
Pkd1 NM_013630.2	s: 5′-CTTCTACTTTGCCCATGAGG as: 5′-CTTCTACTTGCACCTCTGTC	473
Esr1 NM_007956.5	s: 5′-CTCAAGATGCCCATGGAGAG as: 5′-GTTTCCTTTCTCGTTACTGCTG	441
Esr2 NM_207707.1	s: 5′-GACCTACGCAAGACATGGAG as: 5′-CTTGGACTAGTAACAGGGCTG	436
Esrra NM_007953.2	s: 5′-CAGGGCAGTGGGAAGCTAG as: 5′-GCTACTGCCAGAGGTCCAG	362
Esrrb NM_011934.4	s: 5′-GTGGTATCATGGAGGACTCC as: 5′-GTCAATGGCTTTTTAGCAGGTG	388
Esrrg NM_011935.3	s: 5′-CAGCACCATCGTAGAGGATC as: 5′-CATGGCATAGATCTTCTCTGG	442
Gapdh NM_001289726	s: 5′-GTATTGGGCGCCTGGTCAC as: 5′-CTCCTGGAAGATGGTGATGG	200

## Supplementary Materials

The following are available online at https://www.mdpi.com/article/10.3390/ijms22116019/s1. Supplementary Figure S1. CFTR mRNA expression in primary renal epithelial cells. Supplementary Figure S2. YFP-quenching to assess anion permeability. Supplementary Figure S3. Ion currents in M1 mouse collecting duct cells. Supplementary Figure S4. Basal and ATP-induced intracellular Ca^2+^ in primary renal epithelial cells. Supplementary Figure S5. CPA-induced store release in male and female primary renal epithelial cells.
