# Supplementary material for: Gender-Dependent Phenotype in Polycystic Kidney Disease Is Determined by Differential Intracellular Ca2+ Signals
Source: Int J Mol Sci. 2021 Jun 2;22(11):6019. doi: 10.3390/ijms22116019 (PMC8199720; doi:10.3390/ijms22116019)
Supplement: Supplementary file 1 [file ijms-22-06019-s001.zip › ijms-1233102-supplementary.pdf]

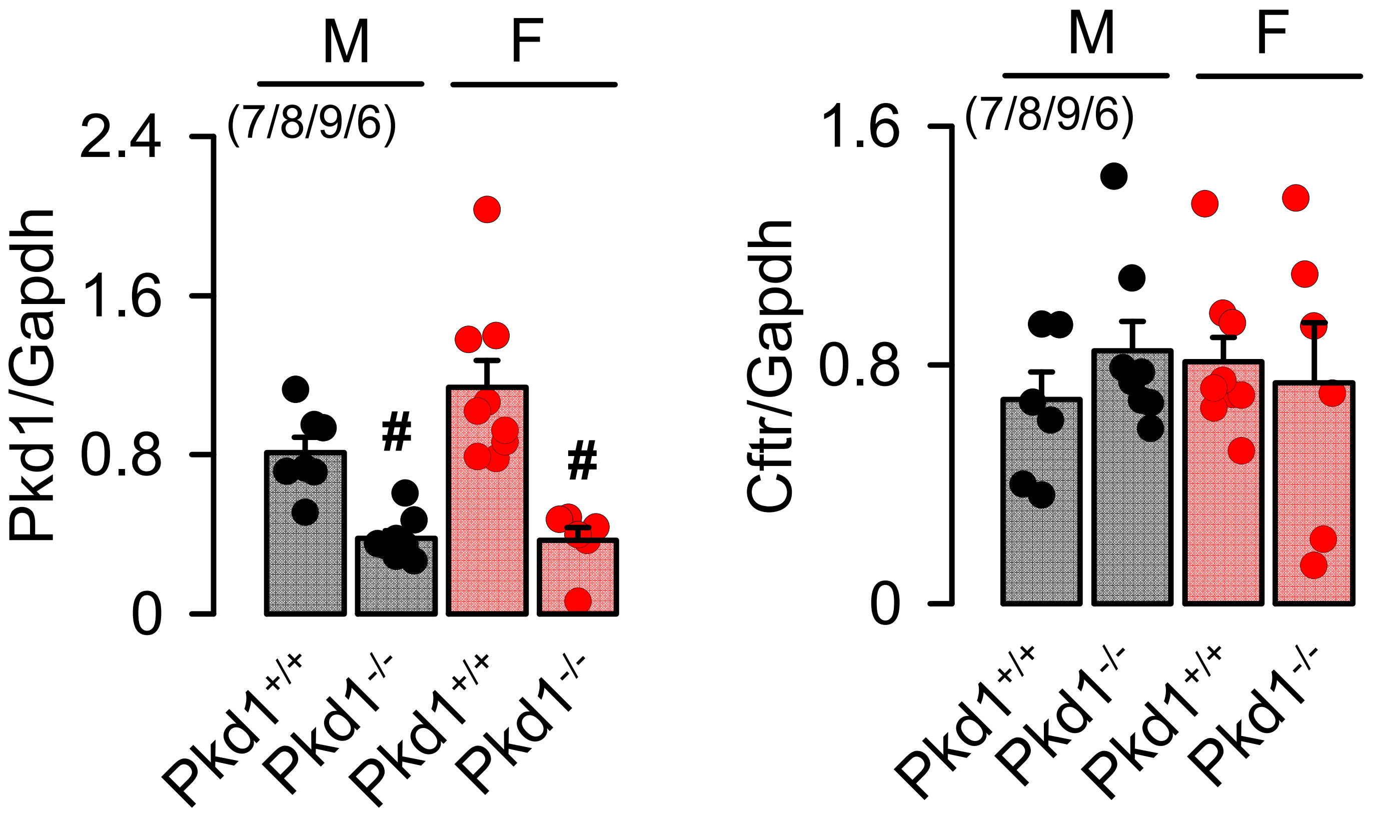

Supplementary Figure S1. *CFTR mRNA expression in primary renal epithelial cells.* Semiquantitative RT-PCR analysis of CFTR mRNA expression in primary medullary renal epithelial cells from male (M) and female (F) Pkd1+/+ and Pkd1-/- mice. Mean ± SEM (number of animals). #indicates significant knockdown of Pkd1 ($p<0.05$; unpaired t-test).

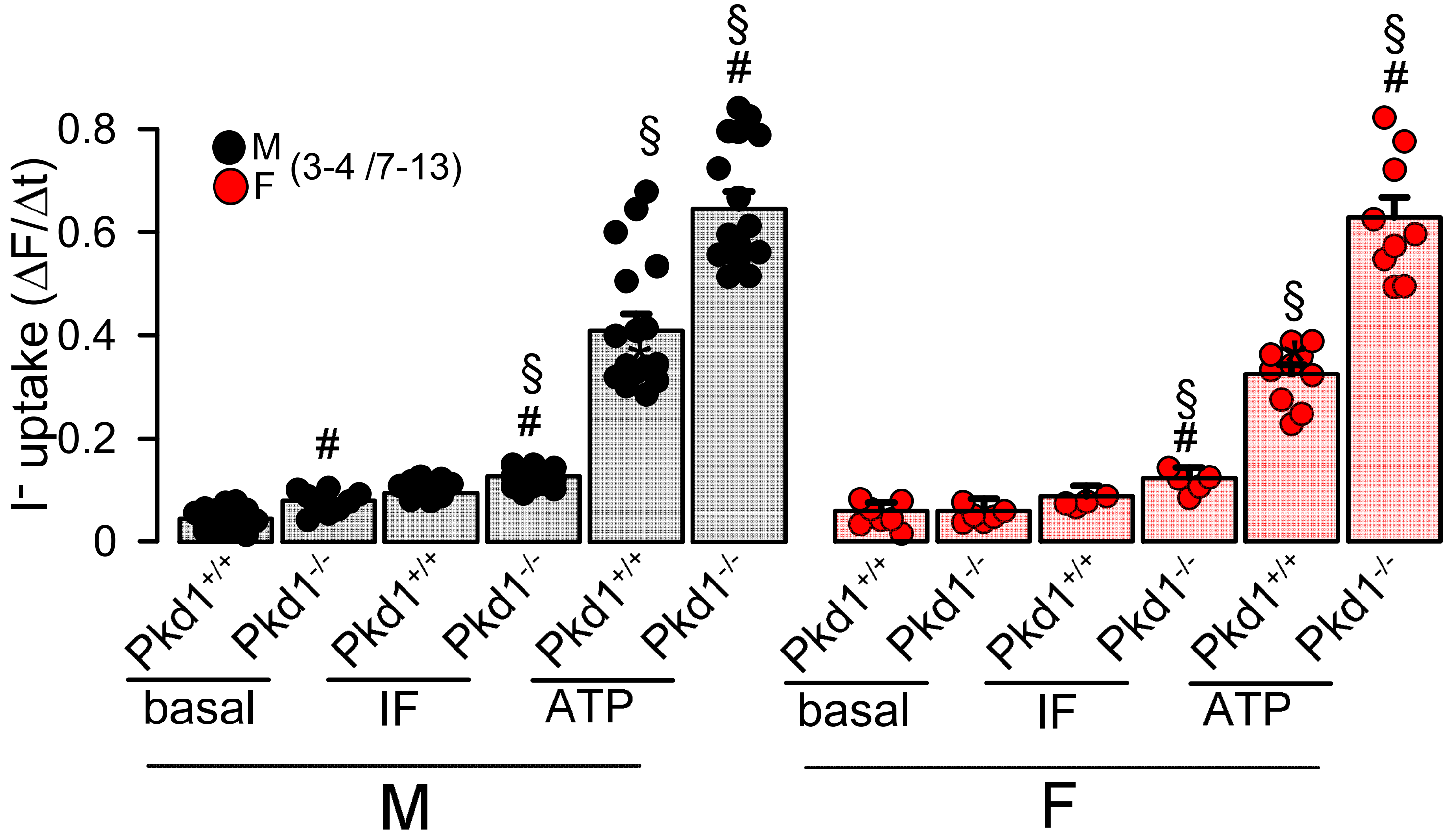

Supplementary Figure S2. *YFP-quenching to assess anion permeability.* Summaries of single cell yellow fluorescence protein (YFP) iodide quenching (I^- uptake) experiments in primary renal epithelial cells from male and female $Pkd1^{+/+}$ and $Pkd1^{-/-}$mice. I^- uptake was assessed under basal conditions and after stimulation with IF (100 µM/2 µM) or ATP (50 µM). Mean ± SEM (number of animals/ number of experiments in each series). #significant difference compared to $Pkd1^{+/+}$ ($p<0.05$; unpaired t-test). §significant difference compared to basal ($p<0.05$; ANOVA and Tukey's post-hoc test).

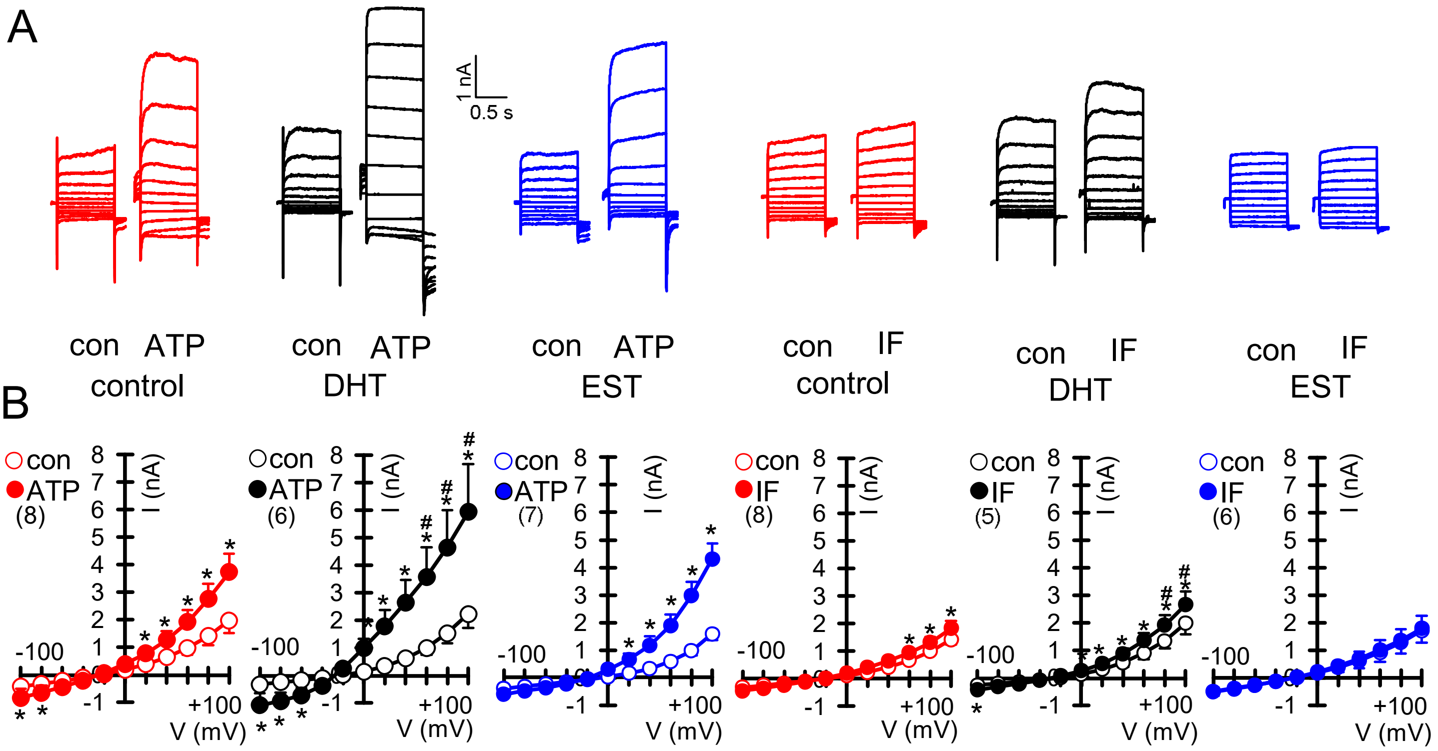

Supplementary Figure S3. *Ion currents in M1 mouse collecting duct cells.* A) Overlay currents for ATP (100 µM) and IBMX/forskolin (IF; 100 µM/2 µM) - activated whole cell currents in untreated cells (control) and cells treated with dihydrotestosterone (DHT; 10 µM, 24 hrs) or estrogen (EST; 10 µM, 24 hrs). B) Summary current / voltage relationships corresponding to overlay currents shown in A. Mean ± SEM (number of experiments). *significant increase by ATP or IF (p<0.05; paired t-test). #significant difference compared to control (p<0.05; ANOVA and Tukey's post-hoc test).

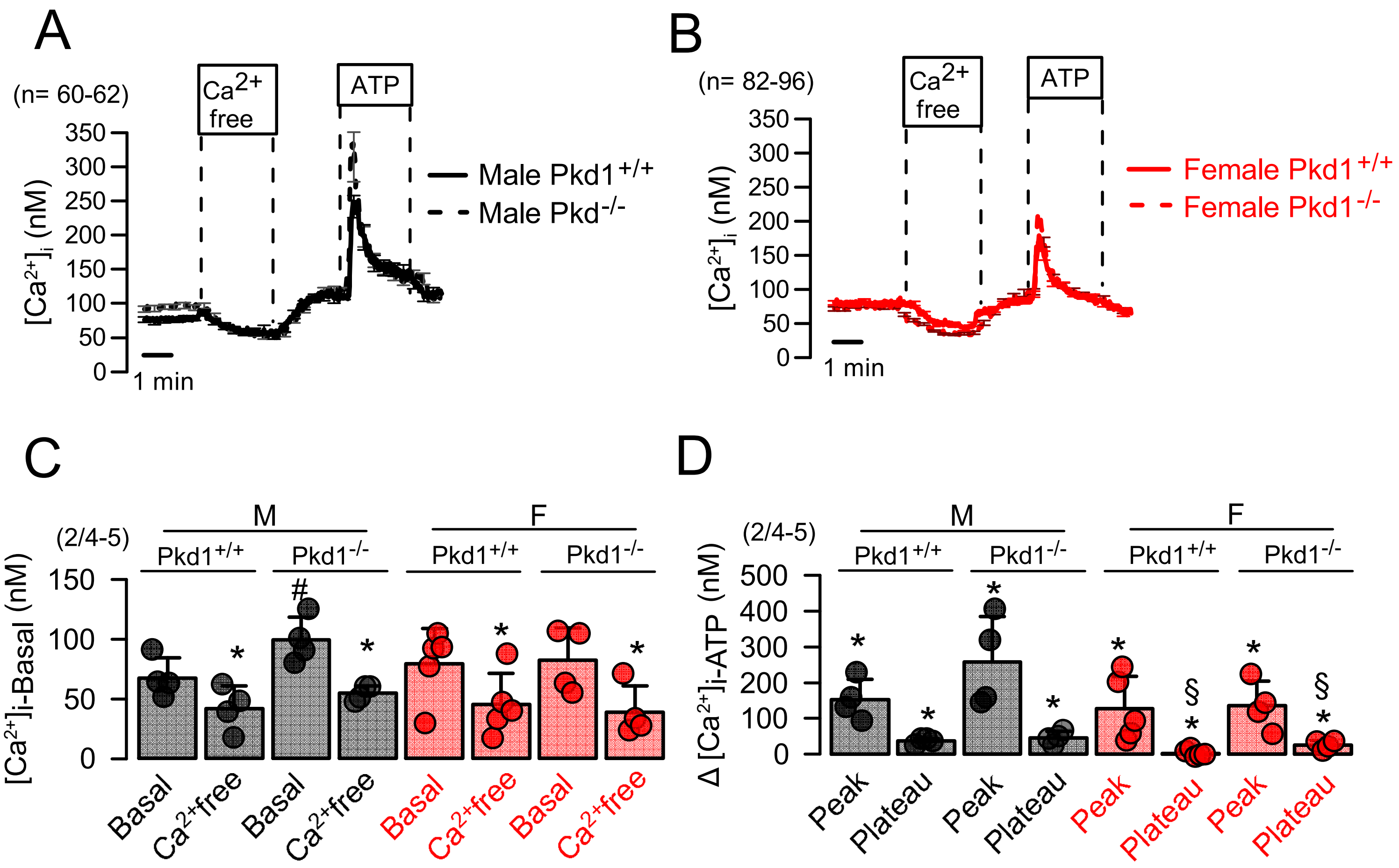

Supplementary Figure S4. *Basal and ATP-induced intracellular Ca^{2+} in primary renal epithelial cells.* A,B) Summary time course for basal and ATP (100 µM) -induced intracellular Ca^{2+} concentrations in male (A) and female (B) Pkd1+/+ and Pkd1-/- mice. C,D) Summary of basal (C) and ATP-induced (D) intracellular Ca^{2+} concentrations in male and female PKD1+/+ and PKD1-/- mice. Mean ± SEM (number of experiments). *significant effect by extracellular Ca^{2+} free and ATP ($p<0.05$; paired t-test). #significant difference compared to Pkd1+/+ ($p<0.05$; ANOVA). §significant difference compared to male ($p<0.05$; ANOVA).

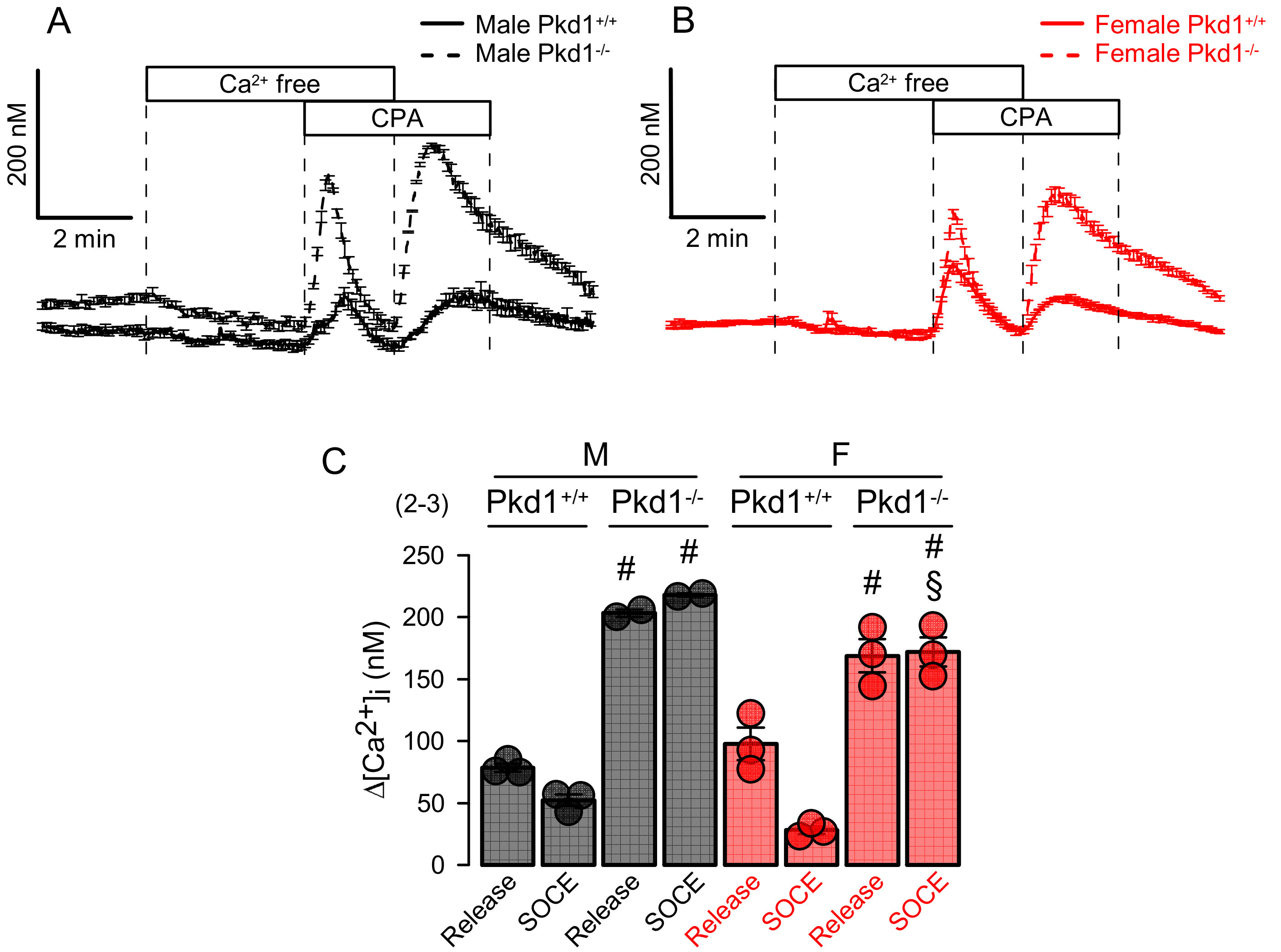

Supplementary Figure S5. *CPA-induced store release in male and female primary renal epithelial cells.* CPA-induced Ca^{2+} store release in primary renal epithelial cells from male (A) and female (B) Pkd1+/+ and Pkd1-/- mice. C) Summary of store release Ca^{2+} and store operated Ca^{2+} entry (SOCE) shown in A and B. Mean ± SEM (number of experiments). #significant difference when compared to Pkd1+/+ ($p<0.05$; unpaired t-test). §significant difference compared to male ($p<0.05$; ANOVA and Tukey's post-hoc test).
